# A New Generation of Porphyrias: A Case of Acute Intermittent Porphyria

**DOI:** 10.7759/cureus.80552

**Published:** 2025-03-14

**Authors:** Mariana Sousa, Francisco Ribeiro, Telma Pais, Sofia Romão, Anabela Oliveira

**Affiliations:** 1 Internal Medicine, Centro Hospitalar Universitário Lisboa Norte - Hospital de Santa Maria, Lisbon, PRT; 2 Nephrology, Centro Hospitalar Universitário Lisboa Norte - Hospital de Santa Maria, Lisbon, PRT

**Keywords:** acute intermittent porphyria, hematin, heme, porphyria, rna interference

## Abstract

Porphyria refers to metabolic disorders caused by dysfunctional heme biosynthesis. Acute intermittent porphyria (AIP) is the most common and severe form of acute porphyria, inherited in an autosomal dominant pattern. During a crisis, diagnosis can be established by collecting urine, plasma, and stool samples for work-up, and treatment should be started.

We report the case of a 41-year-old female patient with a known history of AIP and prior recurrent crises, presenting with severe intracranial hemorrhage due to aneurysm rupture secondary to a hypertensive emergency at the age of 38. She presented to the Emergency Department with nausea, vomiting, abdominal and lower limb pain, left upper and lower limb paresthesias, anxiety, and insomnia. A positive Hoesch test led to a presumptive diagnosis of AIP crisis. Fecal and urinary laboratory work-ups were compatible with an AIP crisis. Genetic studies for new generation porphyrias identified a heterozygous variant p.Leu42Ser in the hydroxymethylbilane synthase (HMBS) gene, probably a pathogenic variant. She completed four days of treatment with hematin, with complete resolution of pain. We highlight the need for prompt evaluation and diagnosis of an AIP crisis, particularly in patients with a known personal or family history of AIP. New therapeutic alternatives with minor side effects are now available and should be started as soon as possible. Given that symptoms are often nonspecific and variable, there should be a high index of suspicion in these patients.

## Introduction

The term porphyria refers to a group of rare metabolic disorders caused by altered activities of enzymes within the heme biosynthetic pathway [1]. Eight enzymes are needed to convert porphyrins into heme, and a deficiency or defect in a specific enzyme required for a particular step in the heme synthesis pathway results in distinct clinical syndromes [1,2]. Although these syndromes have conventionally been classified based on the predominant system involved (cutaneous vs. neurohepatic), they can also be classified as hepatic or erythropoietic, depending on whether pathway intermediates first accumulate in the liver or bone marrow, respectively. However, this classification reflects the pathophysiology but not necessarily the important clinical features of these disorders [3].

Clinically, porphyrias are classified into three categories: acute hepatic porphyrias (AHPs), chronic blistering cutaneous porphyrias, and acute nonblistering cutaneous porphyrias [2]. The three most common porphyrias - porphyria cutanea tarda (PCT), acute intermittent porphyria (AIP), and erythropoietic protoporphyria (EPP) - differ in terms of clinical presentation, diagnostic testing, and treatment [3].

AIP is the most common and severe form of acute porphyria and is inherited in an autosomal dominant pattern with variable penetrance [4]. It is caused by the deficiency of porphobilinogen deaminase (PBGD), also called hydroxymethylbilane synthase (HMBS), the third enzyme in the heme synthesis pathway. This leads to an upregulation of aminolevulinic acid synthase-1 (ALAS1) and the consequent overproduction and accumulation of toxic heme precursors - aminolevulinic acid (ALA) and porphobilinogen (PBG) - in the liver [2]. However, the deficiency alone is not sufficient to cause symptoms. AIP is a multifactorial disorder, and additional factors are required to trigger the appearance of symptoms, such as hormonal changes, the use of certain drugs, excess alcohol consumption, infections, and fasting or dietary changes [4].

This disorder has an overall European prevalence of approximately 1 in 2,000, with a higher incidence in individuals of Northern European descent and a particularly high rate in Sweden (1 in 1,000) due to the founder effect. For this reason, it is also known as Swedish porphyria. Recently, reports of the founder effect have been associated with higher AIP prevalence in certain ethnic groups in Argentina and Spain [3]. Although AIP affects both men and women, women experience more frequent episodes, with longer durations, a greater need for hospitalization, and are more likely to have their first episode at a younger age [3,4].

Symptoms of AIP are often vague and nonspecific, involving multiple organ systems, making diagnosis challenging [3,4]. These episodes develop over the course of several hours or days, and most affected individuals do not experience symptoms between episodes. The most common clinical findings include severe abdominal pain, nausea, vomiting, weakness, constipation, and classic darkened urine discoloration [2]. Unlike most other porphyrias, patients with AIP do not develop cutaneous rashes, as AIP is a hepatic form of porphyria [3].

In this report, we discuss a case of AIP, emphasizing its clinical presentation and management strategies.

## Case presentation

We present a case of a 41-year-old female patient with a known history of AIP, diagnosed at age 10 following family screening (father with known AIP), and no known follow-up until January 2020. At this time, she had complaints of abdominal pain, nausea, vomiting, and new-onset generalized tonic-clonic convulsive crisis. A subarachnoid hemorrhage (SAH) was identified on head CT and confirmed with angiography (focal ectasia in the origin of the left anterior communicating artery). She was admitted to the Neurocritical Intensive Care Unit in a tertiary hospital, undergoing left pterional craniotomy with clipping of the ruptured aneurysm and temporary placement of an external ventricular shunt. A low-carbohydrate diet, started two weeks before, was identified as the trigger for the inaugural AIP crisis - characterized by abdominal pain, dysautonomia, and SAH due to aneurysm rupture secondary to hypertensive emergency. There was a positive Hoesch test, confirming urinary excretion of PBG. Hematin therapy was started at a dose of 3 mg/kg/day (180 mg in a single daily dose for four days), and the patient was later discharged to follow-up consultation at a tertiary hospital with a center for hereditary metabolic diseases.

In December 2022, she presented again to the Emergency Department with persistent complaints of nausea, vomiting, and lower limb pain, with progressive worsening of upper-quadrant abdominal pain over the past week, as well as new-onset anxiety and insomnia. There were no aggravating or mitigating factors.

Upon admission, she was hemodynamically stable, conscious, oriented in space, time, and person, communicative and collaborative, and apyretic. Analytically, there was a serum lipase of 234 U/L (over three times the reference upper limit), with normal liver transaminases, alkaline phosphatase, and bilirubin. She had negative inflammatory markers and normal renal function.

Due to her known AIP history, a Hoesch test was performed with a positive result, and therefore, a presumptive diagnosis of AIP crisis was established. Due to being followed up in consultation at the tertiary hospital with a center for hereditary metabolic diseases and the expected need to carry out targeted therapy with hematin, she was transferred to this hospital. Upon admission, she maintained hemodynamic stability, with pain on right upper quadrant abdominal compression and no pain on decompression. Of the diagnostic tests carried out, there was an unremarkable analytical evaluation, with a normal electrocardiogram, abdominal radiography, and urine exam. She was treated with antiemetic therapy, intravenous hydration with 5% dextrose, and analgesia, with partial pain improvement.

She was subsequently admitted to the Internal Medicine Department. During hospitalization, the patient remained hemodynamically stable, with complete resolution of pain after targeted therapy with hematin (4 mg/kg) for four days, complicated only by phlebitis at the administration site.

The placement of an intrauterine device some days prior was considered as a potential trigger for the crisis. There were no other identifiable precipitating factors for the acute crisis, namely drugs, caloric restriction, infectious conditions, or emotional stress. Urine, fecal, and serological samples were collected for porphyrias study and sent to the reference laboratory.

Regarding the profile of the different urinary porphyrias, it showed changes compatible with AHP, more specifically AIP, due to the finding of an increase in uroporphyrin and coproporphyrin isomer III, together with positive PBG and a delta ALA of 37 umol/mmol (12 times higher than the reference value) (Table 1). Profiles suggestive of AIP were also identified in the fecal sample (Table 2). A panel for new-generation porphyrias (next-generation sequencing) was also carried out, which identified the c-variant as heterozygous 125T>C (p.Leu42Ser) in the HMBS gene. Although the aforementioned variant is not described in the literature, it is probably a “likely pathogenic” variant, according to an analysis carried out using bioinformatics tools.

**Table 1:** Urinary porphyrin profile and biochemical analysis showing changes compatible with acute hepatic porphyria.

Urine analysis	Value (units)	Reference value
Uroporphyrin, octa	1341 nmol/L	≤30
Heptacarboxylporphyrin	45 nmol/L	≤7.0
Hexacarboxylporphyrin	11.2 nmol/L	≤2.0
Pentacarboxylporphyrin	39 nmol/L	≤5.0
Coproporphyrin (isomer I + III)	659 nmol/L	≤110
Coproporphyrin isomer I	117 nmol/L	<110
Coproporphyrin isomer III	542 nmol/L	<110
Delta aminolevulinic acid	37 umol/mmol creatinine	<3.0
Porphobilinogen	Positive	Negative

**Table 2:** Fecal porphyrin profile showing changes compatible with acute hepatic porphyria.

Fecal analysis	Value (units)	Reference value
Uroporphyrin	47.9%	<2%
Coproporphyrin isomer I	35.9%	<75%
Coproporphyrin isomer III	11.2%	<35%
Coproporphyrin isomer III/coproporphyrin isomer II	0.31	<2
Isocoproporphyrin	Negative	Negative

Given the favorable clinical evolution, the patient was discharged from this service on the fifth day. One month after discharge, the patient attended an external consultation with new complaints of nausea, abdominal and lumbar pain, resulting in a diagnosis of a new acute neurovisceral crisis and subsequent admission to the Internal Medicine Department to carry out a new four-day cycle of hematin, with resolution of pain symptoms.

Therefore, it appears that the inaugural crisis of AIP occurred in January 2020, and since then, together with the current hospitalization and according to the consultation records, we count a total of seven episodes of disease flares, with the subsequent completion of six cycles of hematin.

## Discussion

The pathophysiology of porphyrias is closely linked to disruptions in the heme biosynthesis pathway, often caused by gene mutations. In most porphyrias, enzymatic defects lead to the accumulation of toxic intermediates, which cause various clinical symptoms depending on where they accumulate - such as the liver, skin, or nervous system. In AIP, a partial deficiency of PBGD leads to the accumulation of ALA and PBG in the liver, which can manifest as acute neurovisceral symptoms. These intermediates accumulate when the rate-limiting enzyme in heme biosynthesis, delta-ALAS1, is upregulated by factors such as fasting, drugs, or hormonal changes [5].

Environmental triggers are crucial in precipitating AIP attacks, and although the genetic mutation may be present, many patients remain asymptomatic without a triggering event. The exact mechanism of neurotoxicity remains unknown, but accumulating intermediates, particularly ALA, are believed to have direct neurotoxic effects, leading to central, peripheral, and autonomic nervous system dysfunction [2].

AIP manifests primarily as acute neurovisceral symptoms, with abdominal pain being the most common presenting feature. Symptoms include nausea, vomiting, constipation, and motor weakness, and may progress to seizures, coma, and in rare cases, respiratory paralysis. Urine discoloration (dark reddish-brown) is a classic early sign, caused by porphyrin and porphobilin accumulation in urine [6,7].

Diagnosis is challenging due to the nonspecific nature of symptoms. Biochemical testing during an acute attack - specifically measuring urinary PBG and total porphyrins - is essential for diagnosis. The definitive diagnosis of AIP is confirmed by measuring decreased PBGD activity or identifying a pathogenic variant in the HMBS gene. Asymptomatic patients with a family history or suggestive symptoms may benefit from genetic testing and monitoring for potential triggers [1,3].

This case highlights the broad spectrum of AIP manifestations, ranging from potentially fatal intracranial hemorrhages, as seen in this patient’s first crisis, to more minor episodes requiring hematin administration. Although hematin therapy effectively manages AIP crises, it carries risks, including phlebitis and secondary hemochromatosis, especially with recurrent treatment [3,8].

Novel treatments like givosiran, an RNA interference therapeutic targeting hepatic ALAS1 mRNA, offer promising alternatives for patients with refractory AIP. In the ENVISION trial, givosiran significantly reduced the annualized rate of porphyria attacks, lowered the need for hematin, and improved pain management in patients with AHP. However, the treatment comes with potential side effects, including chronic kidney disease and increased liver enzymes, so careful patient selection is essential [9].

The approval of givosiran marks a significant advancement in AIP treatment, providing an alternative for patients with frequent attacks unresponsive to conventional therapy. With continued monitoring of its long-term efficacy and safety, givosiran could reduce the burden of AIP on affected individuals.

## Conclusions

This case of AIP highlights the complexity and variability of its clinical manifestations, ranging from mild neurovisceral symptoms to life-threatening complications, such as SAH. The diagnostic challenge, often due to its nonspecific and fluctuating symptoms, shows the importance of maintaining a high index of suspicion, particularly in patients with a relevant family history. Furthermore, it illustrates the importance of identifying and mitigating potential triggers to prevent recurrent attacks, as well as the crucial role of prompt biochemical testing and targeted therapy.

Given the patient’s multiple disease flares despite conventional therapy, emerging treatments like givosiran offer promising alternatives for recurrent attacks, potentially reducing long-term morbidity and improving quality of life. Continued research into the pathophysiology and long-term outcomes of novel therapies will be essential for the management of this rare but debilitating condition.
